# Revascularization for Posterior Cerebral Artery Infarction in Decompensated Moyamoya Disease

**DOI:** 10.7759/cureus.5681

**Published:** 2019-09-17

**Authors:** Gabe Sexton, Matthew Lommen, Caleb J Heiberger, Tej I Mehta, Douglas Yim

**Affiliations:** 1 Pathology, University of South Dakota Sanford School of Medicine, Sioux Falls, USA; 2 Medicine, University of South Dakota Sanford School of Medicine, Sioux Falls, USA; 3 Radiology, University of South Dakota Sanford School of Medicine, Sioux Falls, USA; 4 Interventional Radiology, Avera McKennan Hospital and University Health Center, Sioux Falls, USA

**Keywords:** moyamoya, infarction, angiography, stenosis

## Abstract

Moyamoya disease is a rare pathological disorder characterized by progressive intracranial artery stenosis and collateral vessel formation. Posterior cerebral artery involvement is rare with a predilection towards infarction. Herein we present a case of a young female with moyamoya disease treated with bilateral encephalomyosynangiosis which subsequently progressed to posterior cerebral artery involvement, requiring encephalomyosynangiosis to prevent further infarction.

## Introduction

Moyamoya disease is characterized by chronic progressive stenosis of large intracranial arteries and subsequent development of an extensive network of small collateral ("moyamoya") vessels. Ischemia may result secondary to steno-occlusive vascular lesions, while the small vessels formed are prone to hemorrhage [1]. Thromboembolism is also thought to play a role, particularly in large infarctions. The rate of admissions due to moyamoya disease in the United States has been estimated at 0.57/100,000 persons/year [2]. The involvement of the posterior cerebral circulation is considered rare, and may represent an independent risk factor for cerebral infarction [3,4]. This case report serves to highlight the treatment and outcome of a rare sequela associated with a rare disorder.

## Case presentation

A 34-year-old Caucasian female with a longstanding history of moyamoya disease underwent bilateral encephalomyosynangiosis using superficial temporal arteries at the age of 12 years. She presented with episodic left-sided hemiparesis and hypesthesia, as well as exacerbation of chronic headache symptoms.

**Figure 1:**
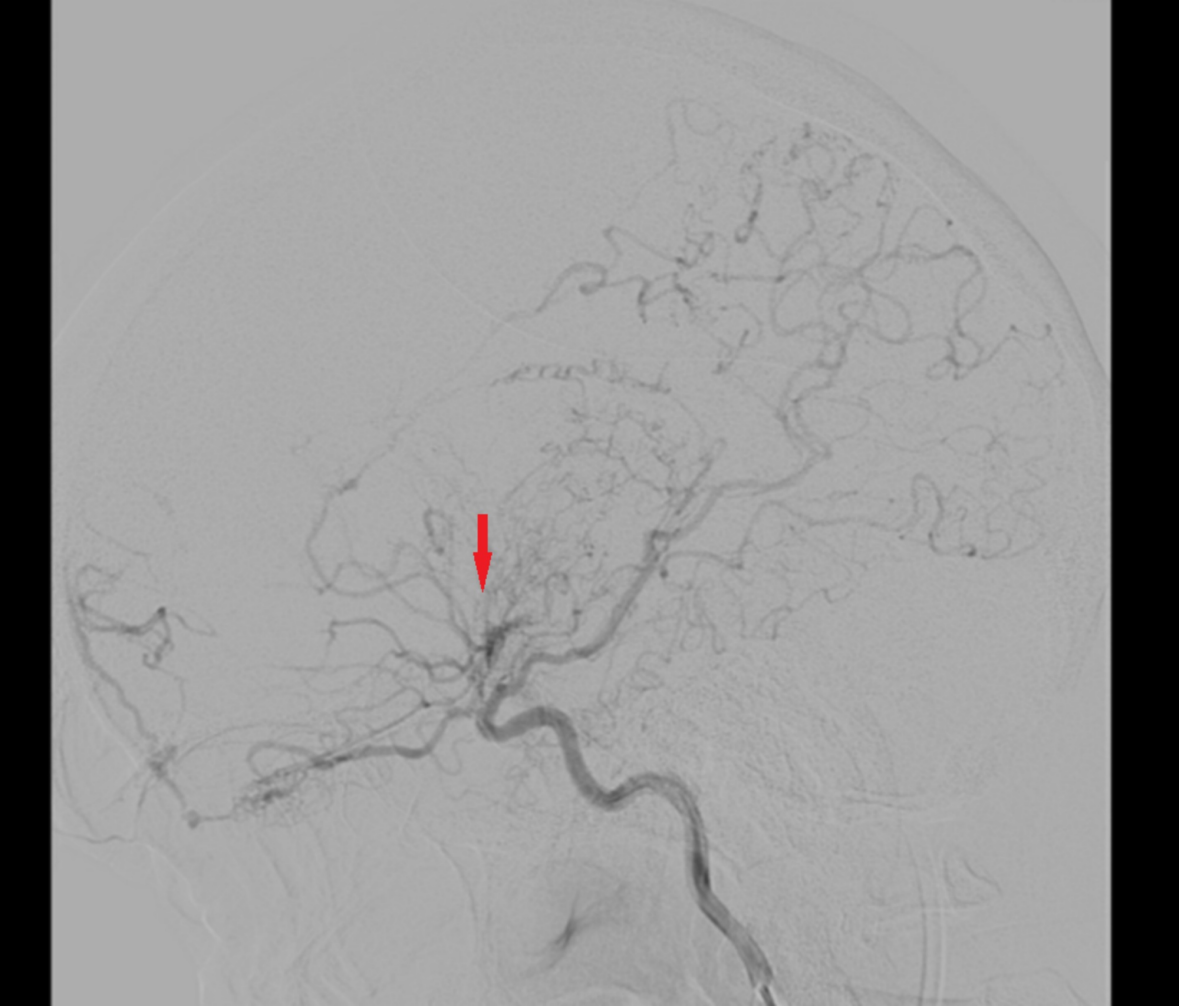
Left-sided cerebral angiogram demonstrating occlusion past the origin of the anterior choroidal artery with collaterals and engorgement of the opthalmic, anterior choroidal, and lenticulostriate arteries (red arrow).

**Figure 2:**
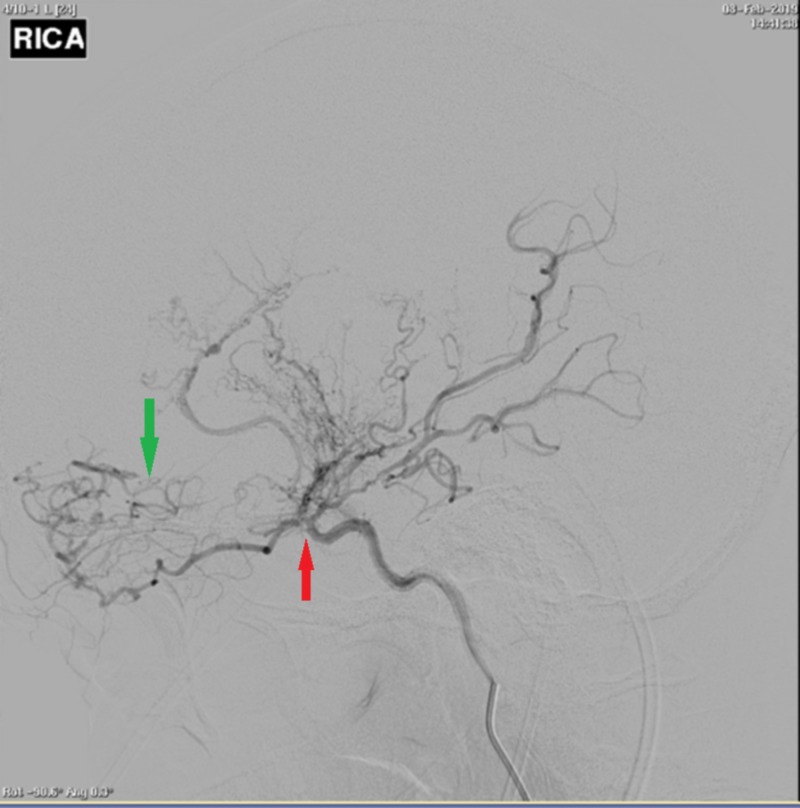
Right-sided cerebral angiogram demonstrating occlusion past the origin of the anterior choroidal artery (red arrow). Proliferation of branches off the anterior choroidal, distal opthalmic, lenticulostriate, and posterior communicating artery is the only source of supply to the hemisphere (green arrow).

Cerebral angiography showed Suzuki stage III moyamoya disease with a reduced blood flow through the patient's right synangiosis as compared to the left (Figures 1, 2).

**Figure 3:**
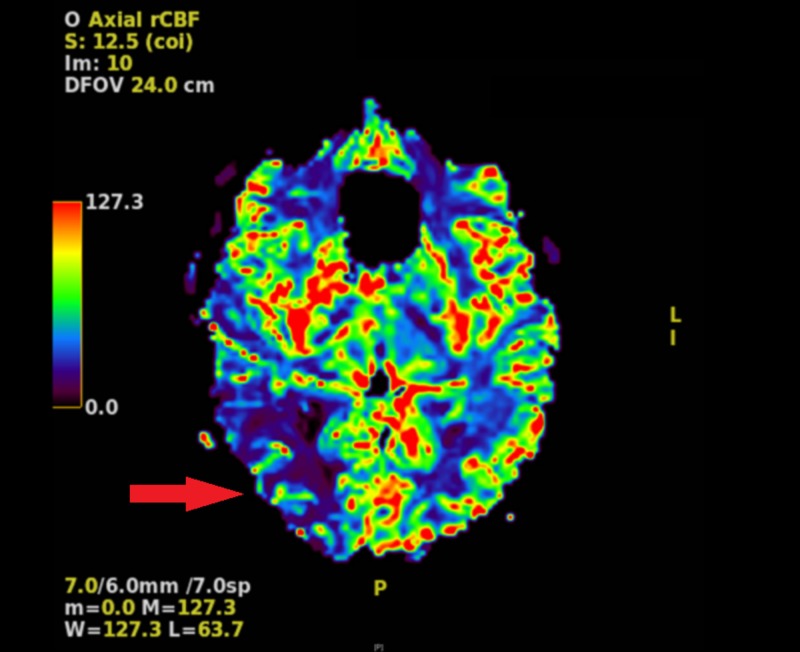
MRI perfusion demonstrating decreased cerebral blood flow in the posterior right hemisphere compared to the left hemisphere (red arrow).

**Figure 4:**
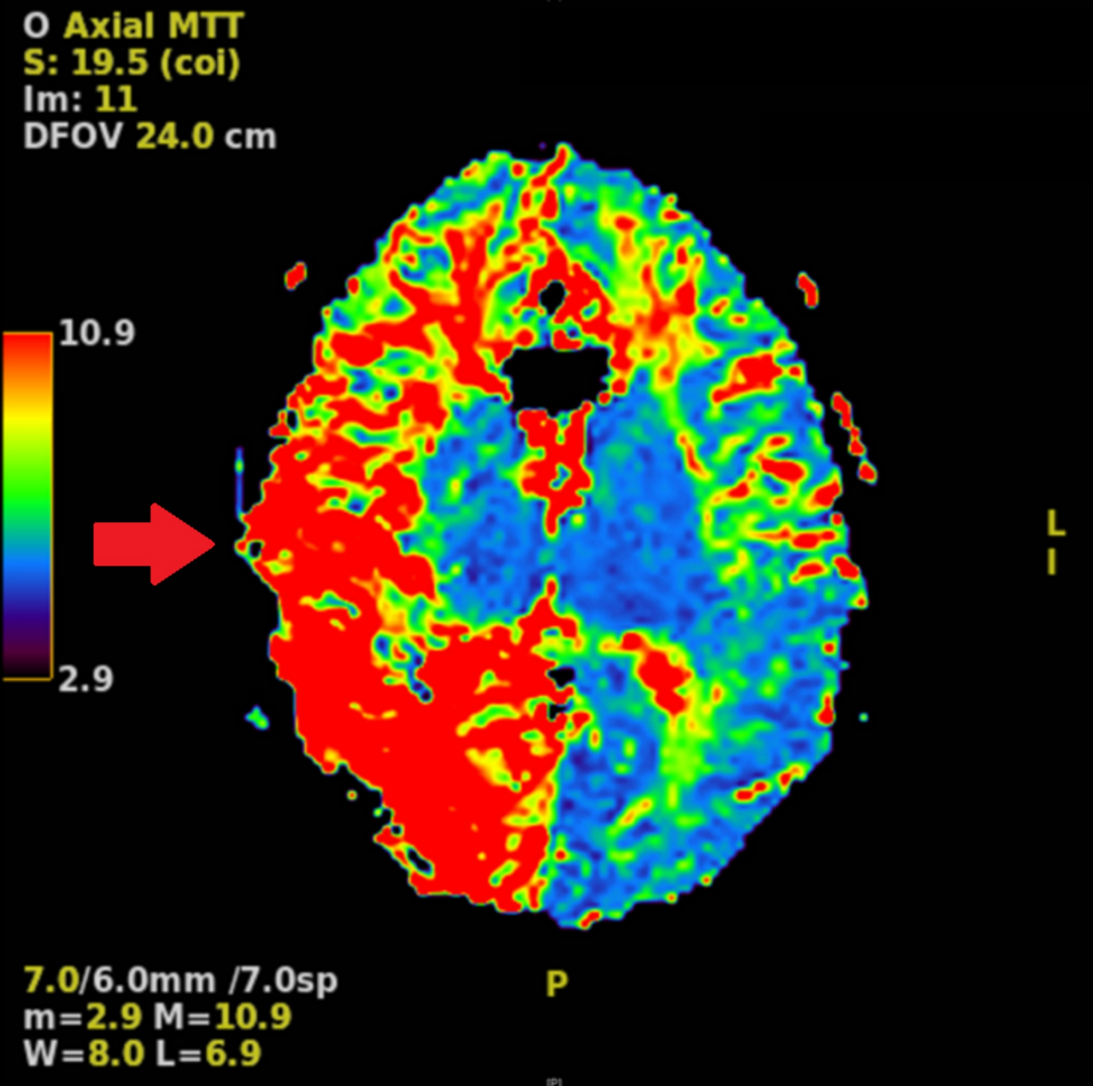
MRI perfusion study demonstrating prolonged mean transit time throughout the right hemisphere relative to the left hemisphere, more predominant posteriorly (red arrow).

Computed tomography (CT) angiography revealed a decrease in blood flow in the right cerebral hemisphere as compared to the left, with no significant flow identified through the right posterior cerebral artery. Magnetic resonance imaging (MRI) demonstrated a region of acute to subacute infarction in the right occipital and parietal lobes. Perfusion studies revealed a decreased cerebral blood flow (CBF) in the posterior part of the right cerebral hemisphere, consistent with the location of the infarction; an increased right hemispheric mean transit time most prominent posteriorly; and an increased right hemispheric cerebral blood volume (CBV), with a coincident reduction of CBV in the area of infarction (Figures 3, 4).

The patient subsequently underwent encephaloduroarteriomyosynangiosis (EDAMS) using the right occipital artery. Surgery was completed without complications, and the patient was discharged home in good condition one day after the procedure.

## Discussion

Diagnosis of moyamoya disease may be made via conventional catheter-based angiography (revealing distal stenosis of the intracranial internal carotid artery [ICA] and the aforementioned network of moyamoya vessels), CT angiography, and MRI. CT angiography is useful for visualizing the intracranial steno-occlusive lesions of moyamoya disease, but MR modalities are able to provide significantly more information regarding flow status and effects on surrounding tissue [1].

A pattern of linear hyperintensity following the sulci on MR fluid-attenuation inversion recovery sequences, known as the "ivy sign," is indicative of a reduction in cortical blood flow due to moyamoya arteriopathy [1,5]. MR angiography may show significant flow voids in the basal ganglia and thalamus (secondary to moyamoya-associated collateral vessels), in combination with reduced flow voids through the internal, middle, and anterior cerebral arteries. Taken together, these findings are nearly diagnostic of moyamoya disease [1].

The grading of moyamoya disease severity is based on the Suzuki staging system, which assigns stages I-VI based on the degree of collateral development and ICA stenosis. Each grade is defined as follows. Grade I: narrowing of the ICA apex; grade II: ICA narrowing with development of collateral moyamoya vasculature; grade III: progression of ICA stenosis with increased numbers of moyamoya collaterals; grade IV: development of external carotid artery (ECA) collaterals; grade V: decreasing moyamoya vessels with intensification of ECA collaterals; Grade VI: complete ICA occlusion with disappearance of moyamoya vessels [1]. Revascularization, such as through EDAMS, with follow-up angiography to monitor perfusion status is the treatment of choice for symptomatic moyamoya disease [4].

Cho et al. found that frontal lobe lesions made up of 64.7% of observed lesions in adults in their 2011 study of moyamoya infarct patterns [6]. A pattern of ischemia and infarction shifting from brain regions supplied by the anterior circulation in less-advanced cases to those regions supplied by the posterior circulation in more advanced cases has also been reported [7].

## Conclusions

Moyamoya disease is characterized by stenosis of large intracranial arteries, primarily affecting the anterior cerebral circulation, and development of characteristic moyamoya collateral vessels. Diagnosis may be confirmed by cerebral angiography, CT angiography, MRI techniques, or some combination of these modalities. The Suzuki staging system is used for assessing severity of moyamoya disease and defines six levels of increasing severity from appearance of isolated ICA stenosis (grade I) to complete ICA occlusion and absence of visualized intracranial moyamoya vessels (grade VI). Follow-up angiograms are recommended following anterior circulation revascularization procedure, and if insufficient CBF is detected in the posterior circulation, a pre-emptive EDAMS utilizing the occipital artery should be considered.
